# Suicide in Schizophrenia: A literature review

**DOI:** 10.1192/j.eurpsy.2023.2365

**Published:** 2023-07-19

**Authors:** M. Huete Naval, R. Galerón Guzmán, E. Herrero Pellón, P. Albarracín Marcos, B. Serván Rendoluna

**Affiliations:** 1 Hospital Clínico San Carlos, Madrid, Spain; 2 Psychiatry, Hospital Clínico San Carlos, Madrid, Spain

## Abstract

**Introduction:**

More than 90 percent of patients who attempt suicide have a psychiatric disorder. The diagnosis of schizophrenia is associated with a decrease in life expectancy of about 10 years, with suicide being the most important related factor. Literature suggests that the risk of suicide death in this population has been found to be 10 to 20 times higher than that in the general population

**Objectives:**

To present a case report of a patient with a first psychotic episode and suicide attempt focusing on clinical features and risk factors.

**Methods:**

Presentation of a clinical case supported by a non-systematic review of literature containing the key-words “suicide”, “Suicidal ideation”, “psychosis” and “schizophrenia”.

**Results:**

This is a case report of a male 28-year-old patient, with no known psychiatric history, admitted to our inpatient service after a suicide attempt by precipitation. In a first evaluation, the patient presented psychotic symptoms consisting of paranoid delusions, auditory hallucinations, tendency to social isolation and the appearance of self-harming ideation in the days prior to the episode. After initiation of antipsychotic medication, a significant improvement in positive symptoms was observed. The patient has since had no delusions or hallucinations and is living independently at home.

Contemporary research studies indicate that the lifetime rate of completed suicide in individuals with schizophrenia is between 4% and 13%. Several specific risk factors have been described in the schizophrenia population, such as early stage of the illness, lack of adherence to treatment, recurrent relapses, comorbid depression and the paranoid subtype. The antipsychotic treatments with the most scientific evidence are clozapine, risperidone, olanzapine and quetiapine. Within psychotherapy, cognitive behavioral therapy appears to be the most effective.

**Conclusions:**

- It is important to know the risk factors that are associated with an increased risk of suicide in patients diagnosed with schizophrenia.
- An early intervention and specific treatment can improve prognosis of this population.

**Disclosure of Interest:**

None Declared

